# Analysis of the relationship between *MIR155HG* variants and gastric Cancer susceptibility

**DOI:** 10.1186/s12876-020-1169-8

**Published:** 2020-01-20

**Authors:** Wenjing Zou, Xu Li, Cheng Li, Dan Liu, Yanyan Lv, Ying Yang, Nan Ye, Dan Guo, Shuixiang He

**Affiliations:** 1 Department of gastroenterology, First Affiliate Hospital of Xi’an JiaoTong University, #227 West Yanta Road, Xi’an, 710061 Shaanxi Province China; 2 Department of The fifth of Internal Medicine, Xi’an No5 Hospital, Xi’an, 710082 Shannxi China; 3 Department of The First of Internal Medicine, Tumor Hospital of Shannxi Province, The Affiliate Hospital of Medical College of Xi’an JiaoTong Univrsity, Xi’an, 710061 Shannxi China; 4 Department of Geriatrics, Xi’an Central Hospital, Xi’an, 710003 Shannxi China; 5 Department of Rheumatology, Xi’an No5 Hospital, Xi’an, 710082 Shannxi China; 6 Department of The Second of Internal Medicine, Xi’an No5 Hospital, Xi’an, 710082 Shannxi China

**Keywords:** Gastric cancer, *MIR155HG*, Single nucleotide polymorphism, Case-control study

## Abstract

**Background:**

Gastric cancer is one of the most common cancers in the world and a major cause of cancer-related death. This study aims to determine whether genetic variations in *MIR155HG* could be associated with gastric cancer risk.

**Materials & methods:**

A total of 506 gastric cancer patients and 500 healthy controls were enrolled in this study. Genotypes were examined with the MassARRAY platform and data management and analysis were conducted with the Typer Software. Odds ratios (OR) and 95% confidence intervals (CIs) were calculated with logistic regression adjusting for age and gender to evaluate the associations between SNPs with gastric cancer in genetic model analysis.

**Results:**

The “CC” genotype of rs4143370 decreased the risk of gastric cancer in genotype model (*p* = 0.020) and recessive model (*p* = 0.018). Inversely, the “CC” genotype of rs1893650 increased the risk of gastric cancer in genotype model (*p* = 0.023) and recessive model (*p* = 0.014). Stratified analysis showed that rs11911469 was associated with an increased risk of gastric cancer only among the male group in the dominant model (*p* = 0.039) and additive model (*p* = 0.030). The haplotype analysis showed a strong linkage disequilibrium among these six SNPs (rs4143370, rs77699734, rs11911469, rs1893650, rs34904192 and rs928883).

**Conclusion:**

This study confirmed the relationship between SNPs of *MIR155HG* and the gastric cancer risk among the Chinese Han population. Our data may provide a new perspective to understand the aetiology of gastric cancer.

## Background

Gastric cancer is rampant all over the world, which brings serious living burden and economic pressure to human beings. Gastric cancer was reported to be the third leading cause of cancer-related mortality [1]. The incidence of this cancer varies by region and ethnicity. In East Asia, the incidence and mortality of gastric cancer are the highest, especially in China [2]. Gastric cancer is a complex multifactorial disease and its pathogenesis is still obscure. Epidemiological studies have demonstrated that interaction of environmental factors and genetic factors has been found to contribute to the risk of gastric cancer. Studies have shown that the first-degree relatives of gastric cancer patients tend to have about 1.3 to 3.0 fold higher relative risk for gastric cancer than those without relatives with gastric cancer [3].

MicroRNAs (miRNAs) are a class of important non-coding RNAs that play a biological role by inhibiting the expression of their targets at the post-transcriptional level. MiRNAs are involved in a variety of cellular processes, from embryonic development and immunity to carcinogenesis, by interacting with their multiple targets [4]. MiRNAs also play a vital role in the pathogenesis of gastric cancer [5, 6]. MicroRNA-155 (miR-155) is a multifunctional miRNA that is involved in many disease processes including hematopoietic development [7, 8], inflammatory responses [9, 10], autoimmune [11], and tumorigenesis [12, 13]. However, the function of miR-155 in gastric cancer has not been fully elucidated. MiR-155 is a transcription product of its host gene (*MIR155HG*), and its expression may be affected by genetic variations of the *MIR155HG* gene.

Single nucleotide polymorphisms (SNPs) are the most common types of genetic variations that affect the risk of disease by altering the expression of related genes. To our knowledge, there were three studies have reported the relationship between *MIR155HG* SNPs and multiple sclerosis [14], atopic eczema [15], and epilepsy [16]. However, the exact relation between *MIR155HG* SNPs and gastric cancer risk is still undetermined.

Therefore, in this study, we conducted a case-control study among the Chinese Han population to clarify the relationship between *MIR155HG* SNPs and the risk of gastric cancer. Our results will help to understand the occurrence and development of gastric cancer from the perspective of molecular genetics, thus providing a theoretical basis for the early detection of gastric cancer.

## Methods

### Subjects

A total of 506 gastric cancer patients and 500 healthy controls were recruited at the Shaanxi Provincial Cancer Hospital. All the individuals were genetically unrelated ethnic northwest Han Chinese and all patients were diagnosed with gastric cancer by pathological analysis. We excluded the patients who received palliative surgery, preoperative treatment, or with incomplete pathological data. Subjects had no history of hereditary or malignant diseases. The clinical features of patients were collected from the patients’ medical records provided, including age, gender, and clinical indicators. All individuals were informed of the purpose and experimental procedures of the study.

### DNA extraction and SNP genotyping

Peripheral blood samples (5 mL) were collected from each subject. Genomic DNA was extracted from blood samples using the GoldMag-Mini Purifcation Kit (GoldMag Co. Ltd., Xi’an, China). The concentration and quality of the DNA were measured on a Nanodrop 2000 spectrophotometer (Thermo Scientific, USA). Seven SNPs with minor allele frequencies (MAFs) > 5%, referenced from the 1000 Genomes database (*http://www.internationalgenome.org/*), were selected for subsequent analysis. Agena MassARRAY Assay Design Software (version 3.0, Agena Bioscience, San Diego, CA, USA) was used to design multiplexed SNP MassEXTEND assay. Agena MassARRAY RS1000 was used to detect SNP genotyping. Data management and analysis were conducted using Agena Software (version 4.0, Agena, San Diego, CA, USA) [17, 18].

### Statistical analyses and bioinformatics analysis

The Hardy-Weinberg Equilibrium (HWE) was tested by using Fisher’s exact test in the healthy control group. The odds ratios (ORs), 95% confidence intervals (CIs) and *p*-values were calculated with the logistic regression model to assess the risk of gastric cancer. Four models (genotype, dominant, recessive, and additive) were used to assess the association between each genotype and the risk of gastric cancer [19]. The Haploview software package (version 4.2) and SHEsis software platform were used to analyze the pairwise linkage disequilibrium (LD), haplotype construction, and genetic association of polymorphism loci. SPSS software (version 22.0; SPSS Inc., Chicago, IL, USA) was used for all statistical analyses. All *p*-values were two-sided, and the adjusted *p*-value of less than 0.05 was considered to be significant.

HaploReg v4.1 (*https://pubs.broadinstitute.org/mammals/haploreg/haploreg.php*) was employed to predict the potential functions of the candidate SNPs.

## Results

### Demographic and clinical characteristics

The characteristics of gastric cancer patients shown in Table 1. The total of 506 patients (380 males, 126 females) with the average age of 61.11 ± 0.50 and 500 age-matched healthy people (376 males, 124 females) with the mean age of 60.31 ± 0.44 were enrolled. There was no significant difference in age and gender distribution between the case and the control group (*p* > 0.05). In addition, the variance analysis of all the clinical indicators among patients with the different genotypes of selected SNPs shown in the Additional file 1: Table S1.

**Table 1:** Characteristics of the study population

	Cases	Controls	*p*
Total	506	500	
Age			0.229^a^
Mean ± SD	61.11 ± 0.50	60.31 ± 0.44	
Sex			0.970^b^
Male	380 (75%)	376 (75%)	
Female	126 (25%)	124 (25%)	
Stage			
I-II	105 (21%)		
III-IV	238 (47%)		
Absence	163 (32%)		
LNM			
Negative	234 (46%)		
Positive	96 (19%)		
Absence	176 (35%)		
CEA (0-20 ng/ml)			
Mean ± SD	16.82 ± 10.21		
SF (20-240 ng/ml)			
Mean ± SD	89.05 ± 8.19		
TNF (0.74–1.74fmol/ml)			
Mean ± SD	1.01 ± 2.08		
CA-50 (0-25 U/ml)			
Mean ± SD	6.92 ± 11.30		
CA-199 (0-37 U/ml)			
Mean ± SD	39.70 ± 76.36		
CA-242 (0-20KU/ml)			
Mean ± SD	13.85 ± 27.57		
AFP (0-20 ng/ml)			
Mean ± SD	12.08 ± 15.33		

*SD* standard deviation, *LNM* lymph node metastasis, *CEA* carcino-embryonic antigen, *SF* serum ferritin, *TNF* tumor necrosis factor, *CA* carbohydrate antigen, *AFP* alpha-fetoprotein

^a^ *p* values were calculated from independent sample *t*-test

^b^ *p* values were calculated from two-sided χ^2^ test

### The basic information of selected SNPs

The basic information of the seven SNPs in *MIR155HG* shown in Table 2, including gene, chromosome, position, alleles, MAF in cases and controls and functional effects. All these SNPs complied with Hardy-Weinberg equilibrium in the control group. Additionally, we used HaploRegv4.1 to annotate the functional elements containing these selected SNPs. The results showed that the SNPs of *MIR155HG* were involved in the regulation related to DNAase, mark the promoter histone, change motifs, GRASP QTL hits, enhance histones, and bind proteins, suggesting they might exert biology functions in these ways in gastric cancer.

**Table 2:** Basic information of candidate single nucleotide polymorphism (SNPs) in the study

SNP ID	Genes	Chr.	Position	Alleles A/B	MAF		*p*-HWE^a^	*p*^**b**^	OR(95%CI)	HaploReg
					Case	Control				
rs4143370	MIR155HG	chr21	25,564,661	C/G	0.156	0.175	0.087	0.255	0.87 (0.69–1.10)	Promoter histone marks, Enhancer histone marks, DNAse, Proteins bound, Motifs changed, Selected eQTL hits
rs77218221	MIR155HG	chr21	25,565,063	C/T	0.042	0.056	0.662	0.131	0.73 (0.48–1.10)	Promoter histone marks, Enhancer histone marks, Motifs changed
rs77699734	MIR155HG	chr21	25,566,995	C/G	0.096	0.092	0.293	0.778	1.04 (0.77–1.41)	Promoter histone marks, Enhancer histone marks, DNAse, Proteins bound
rs11911469	MIR155HG	chr21	25,567,971	A/C	0.127	0.103	0.339	0.089	1.27 (0.96–1.67)	Promoter histone marks, Enhancer histone marks, DNAse, Proteins bound, Motifs changed
rs1893650	MIR155HG	chr21	25,568,503	C/T	0.189	0.174	0.535	0.391	1.10 (0.88–1.39)	Promoter histone marks, Enhancer histone marks, DNAse, Proteins bound, Motifs changed, GRASP QTL hits, Selected eQTL hits
rs34904192	MIR155HG	chr21	25,569,623	A/G	0.252	0.266	0.423	0.473	0.93 (0.76–1.14)	Promoter histone marks, Enhancer histone marks, DNAse, Proteins bound, Motifs changed, Selected eQTL hits
rs928883	MIR155HG	chr21	25,571,713	A/G	0.435	0.460	0.785	0.256	0.90 (0.76–1.08)	Enhancer histone marks, DNAse, Proteins bound, Motifs changed, Selected eQTL hits

*SNP* single nucleotide polymorphism, *MAF* minor allele frequency, *HWE* Hardy-Weinberg equilibrium, *OR* odds ratio, *95% CI* 95%confidence interval

^a^ HWE *p*^a^-value were calculated from Fisher’s exact test

^b^ *p* values were calculated from two-sided χ^2^ test

### SNPs and the risk of gastric cancer

In Table 2, there was no significant association in allele frequency between cases and controls compared by χ2 test. Then, four inheritance models (genotype model, dominant model, recessive model, and additive model) were applied for analysing the association between each SNP and gastric cancer risk by logistic regression analysis adjusted for age and gender (Table 3). The result indicated that the “CC” genotype of rs4143370 was associated with a decreased risk of gastric cancer in genotype model (OR = 0.37, 95% CI = 0.16–0.86, *p* = 0.020) and recessive model (OR = 0.37, 95% CI = 0.16–0.85, *p* = 0.018), respectively. Inversely, the “CC” genotype of rs1893650 was associated with an increased risk of gastric cancer in genotype model (OR = 2.01, 95% CI = 1.10–3.65, *p* = 0.023) and recessive model (OR = 2.10, 95% CI = 1.16–3.80, *p* = 0.014).

**Table 3:** Analysis of association between rs4143370 and rs1893650 polymorphism and risk of gastric cancer

SNP ID	Model	Genotype	Case(*N*)	Control(*N*)	Crude		Adjusted^a^	
					OR(95%CI)	*p*^**†**^	OR(95%CI)	*p*^**†**^
rs4143370	Genotype	GG	356	346	1			
		GC	142	133	1.04 (0.79–1.37)	0.795	1.04 (0.79–1.37)	0.794
		CC	8	21	0.37 (0.16–0.85)	0.019*	0.37 (0.16–0.86)	0.020*
	Dominant	GG	356	346	1		1	
		GC-CC	150	154	0.95 (0.72–1.24)	0.690	0.94 (0.72–1.24)	0.698
	Recessive	GG-GC	498	479	1		1	
		CC	8	21	0.37 (0.16–0.84)	0.017*	0.37 (0.16–0.85)	0.018*
	Additive	–	–	–	0.87 (0.69–1.10)	0.258	0.88 (0.69–1.11)	0.266
rs1893650	Genotype	TT	350	343	1			
		TC	121	140	0.85 (0.64–1.13)	0.254	0.85 (0.64–1.13)	0.261
		CC	35	17	2.02 (1.11–3.67)	0.021*	2.01 (1.10–3.65)	0.023*
	Dominant	TT	350	343	1		1	
		TC-CC	156	157	0.97 (1.75–1.27)	0.845	0.97 (0.75–1.27)	0.852
	Recessive	TT-TC	471	483	1		1	
		CC	35	17	2.11 (1.17–3.82)	0.014*	2.10 (1.16–3.80)	0.014*
	Additive	–	–	–	1.09 (0.88–1.35)	0.419	1.09 (0.88–1.35)	0.421

^a^Adjusted for age and sex in a logistic regression model

^†^*p* values were calculated from wald test

*Bold values indicate statistical significance (*p* < 0.05)

Stratified analysis by gender revealed the relationship of *MIR155HG*-rs11911469 with gastric cancer risk and the results were exhibited in Table 4. We found that rs11911469 was associated with an increased risk of gastric cancer only in the male group under the dominant model (OR = 1.45, 95% CI = 1.02–2.07, *p* = 0.039) and additive model (OR = 1.45, 95% CI = 1.04–2.02, *p* = 0.030).

**Table 4:** Stratification analysis of the association of *MIR155HG* polymorphisms with gastric cancer by gender

SNP ID	Model	Genotype	Male		Female	
			OR(95%CI)	*p*^**†**^	OR(95%CI)	*p*^**†**^
rs11911469	Genotype	AA	1			
		AC	1.42 (0.99–2.03)	0.058	0.86 (0.48–1.55)	0.626
		CC	2.66 (0.51–13.85)	0.245	1.93 (0.17–12.62)	0.595
	Dominant	AA	1		1	
		AC-CC	1.45 (1.02–2.07)	0.039*	0.89 (0.51–1.60)	0.713
	Recessive	AA-AC	1		1	
		CC	2.48 (0.48–12.88)	0.280	1.99 (0.18–22.29)	0.576
	Additive	–	1.45 (1.04–2.02)	0.030*	0.95 (0.55–1.61)	0.835

^†^*p* values were calculated from wald test

*Bold values indicate statistical significance (*p* < 0.05)

### Haplotype analysis

**Fig. 1:**
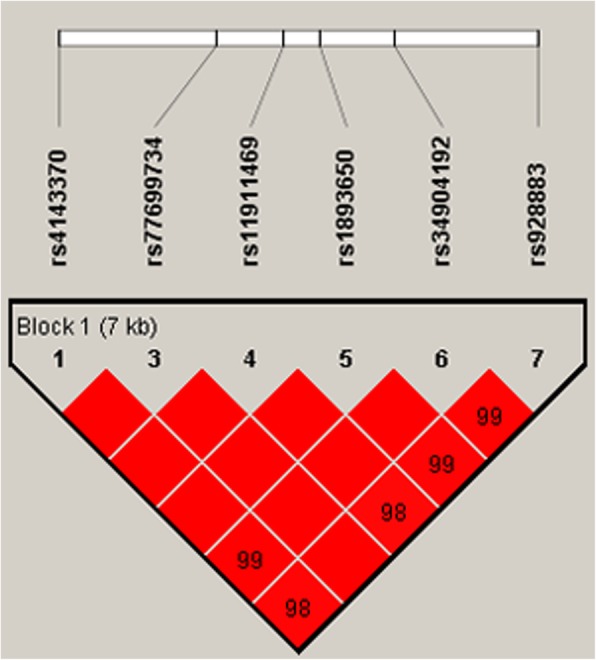
Haplotype block map for the SNPs in *MIR155HG* gene. The numbers inside the diamonds indicate the D’ for pairwise analyses

The haplotype association analysis was based on six SNPs of *MIR155HG* (rs4143370, rs77699734, rs11911469, rs1893650, rs34904192 and rs928883) (Table 5). We observed strong linkage disequilibrium among these six SNPs (Fig. 1). Whereas the results did not provide any statistical evidence of the associations between the haplotypes and gastric cancer risk in the subsequent analysis (all *p*-values were greater than 0.05).

**Table 5:** Haplotype frequencies of *MIR155HG* SNPs and the association with gastric cancer

Haplotype	Haplotype Frequency		OR(95%CI)	*p*^**†**^
	Case	Control		
rs4143370|rs77699734|rs11911469|rs1893650|rs34904192|rs928883				
_GCATGA_	0.568	0.548	1.09 (0.91–1.29)	0.367
_GGATAG_	0.904	0.910	0.94 (0.70–1.27)	0.689
_CCATAG_	0.156	0.174	0.88 (0.70–1.12)	0.298
_GCACGG_	0.189	0.172	1.11 (0.89–1.37)	0.353
_GCCTGG_	0.874	0.896	0.80 (0.60–1.06)	0.116

*OR* odds ratio, *95%CIs* 95% confidence intervals

^†^ *p* values were calculated by logistic regression with adjustment for age and gender

## Discussions

In the present study, selected SNPs in *MIR155HG* and their association with gastric cancer were investigated for the first time. Three *MIR155HG* variants (rs4143370, rs1893650, and rs11911469) were associated with the risk of gastric cancer among the Chinese Han population.

*MIR155HG* was found to be a marker of early stage cancer development [20]. *MIR155HG* gene is activated by MYB transcription factor and thus up-regulated, which in turn leaded to down-regulation of many tumor suppressor genes [21]. Previous studies have shown that three SNPs (rs2829803, rs2282471, rs2829806) of *MIR155HG* were associated with the risk of multiple sclerosis, and two SNPs (rs969885, rs987195) of *MIR155HG* in epilepsy were identified as the genetic susceptibility factors [14, 16]. In this study, three novel SNPs (rs4143370, rs1893650, and rs11911469) were found to significantly affect the gastric cancer susceptibility. Our results suggest a great potential of *MIR155HG* in the early diagnosis of gastric cancer. For many years, intron sequences have been considered essentially non-functional. However, subsequent study showed that intron-containing genes presented higher levels of transcription when compared to intron-less genes in mammalian cells [22], suggesting that introns may be enhancers of transcription. In addition, we hypothesized that the genetic variation of *MIR155HG* gene may influence the expression of its transcription product miR-155, thus affecting its function in tumors. Stratified analysis found that *MIR155HG*-rs11911469 was associated with an increased risk of gastric cancer only in the male group. This result may be due to the presence of some gender-specific genes in the target genes of miR-155. But these internal connections need to be verified by subsequent functional experiments.

In recent years, a large number of studies have shown that miR-155, as an oncomiR, plays a vital role in carcinogenesis. Upregulated mir-155 levels have been found in many solid malignancies [23]. In colorectal cancer, the expression of miR-155-5p was upregulated. It has been proved that high expression of miR-155-5p promoted the proliferation, invasion and metastasis of colorectal cancer cells [24]. In addition, upregulation of mir-155 has been reported to be associated with poor prognosis in lung cancer and breast cancer [25, 26]. In contrast, several studies have revealed that mir-155 may act as a tumor-suppressor in some cancers. Mir-155 expression was down-regulated in melanoma and ovarian cancer and significantly inhibited tumor growth in vivo and in vitro [27, 28]. In addition, Wang et al. found that deficiency mir-155 significantly promoted tumor growth and metastasis in bone marrow-derived inhibitory cells [29]. Similarly, up to now, the reports about miR-155 expression and its role in gastric cancer have been inconsistent. Previous studies reported that the expression of miR-155 was significantly up-regulated and acted as a tumor-promoter in gastric cancer, while another literature suggested that miR-155 was one of the most downregulated miRNAs, which may play a role in inhibiting tumor in gastric cancer [30, 31]. In addition, mir-155 levels were found to be lower in patients with advanced gastric cancer and higher in patients with early gastric cancer [32]. These inconsistent conclusions may be caused by different sources of experimental samples and need to be verified in the future. Although the detailed mechanism of mir-155 in gastric cancer remains to be further explored, it may be a suitable choice for the early diagnosis of gastric cancer patients as a biomarker.

However, we considered that there are some limitations in this study. First, this study was conducted only in the Chinese Han population. Due to differences in regional environment and genetic background, the function of these SNPs in other ethnic groups is worth considering. Second, gastric cancer is a complex disease affected by a variety of inherited and environmental factors. We should not ignore the influence of the interaction between genetic polymorphism and environmental factors on gastric cancer susceptibility.

## Conclusions

To sum up, our study confirmed the relationship between SNPs of *MIR155HG* and the risk of gastric cancer among the Chinese Han population. We hope that this study can provide a new and promising strategy for the early detection of gastric cancer and provide the possibility for the early prevention of this disease. Further studies will increase the sample size and clinical data, as well as carry out functional studies and repeated in different ethnic groups.

## Supplementary information

**Additional file 1: Table S1.** Variance analysis of clinical characteristics among patients and different genotypes of SNPs

## Data Availability

The datasets used and analyzed in the current study are available from the corresponding author on reasonable request.

## Abbreviations

CI: Confidence interval
HWE: Hardy-Weinberg Equilibrium
LD: Linkage disequilibrium
MAF: Minor allele frequency
miRNA: MicroRNA
OR: Odds ratios
SNP: Single nucleotide polymorphism
