# Supplementary material for: Analysis of the relationship between MIR155HG variants and gastric Cancer susceptibility
Source: BMC Gastroenterol. 2020 Jan 20;20:17. doi: 10.1186/s12876-020-1169-8 (PMC6972026; doi:10.1186/s12876-020-1169-8)
Supplement: Supplementary file 1 — Additional file 1: Table S1. Variance analysis of clinical characteristics among patients and different genotypes of SNPs [file 12876_2020_1169_MOESM1_ESM.docx]

**Supplementary Table 1 Variance analysis of clinical characteristics among patients and different genotypes of SNPs.**

| **characteristics** | **rs4143370** | | | |  | **rs77699734** | | | |
| --- | --- | --- | --- | --- | --- | --- | --- | --- | --- |
|  | **CC** | **CG** | **GG** | ***p***^†^ |  | **CC** | **CG** | **GG** | ***p***^†^ |
| CEA (ng/ml) | 15.20 ± 1.12 | 16.10 ± 0.90 | 17.12 ± 0.73 | 0.707 |  | 16.32 ± 0.58 | 19.19 ± 1.85 | 14.48 ± 2.91 | 0.152 |
| SF (ng/ml) | 93.85 ± 2.53 | 89.43 ± 0.87 | 88.82 ± 0.54 | 0.419 |  | 88.51 ± 0.47 | 91.31 ± 1.29 | 91.303 ± 3.05 | 0.054 |
| TNF (fmol/ml) | 1.32 ± 0.43 | 0.89 ± 0.01 | 1.01 ± 0.11 | 0.252 |  | 0.89 ± 0.00 | 1.54 ± 0.63 | 0.90 ± 0.45 | 0.096 |
| CA-50 (U/ml) | 6.87 ± 1.83 | 5.97 ± 1.18 | 7.28 ± 0.75 | 0.657 |  | 6.71 ± 0.66 | 8.08 ± 1.83 | 2.77 ± 1.75 | 0.578 |
| CA-199 (U/ml) | 25.27 ± 3.53 | 30.56 ± 5.13 | 43.38 ± 5.79 | 0.406 |  | 40.43 ± 4.63 | 37.53 ± 13.13 | 16.27 ± 4.97 | 0.842 |
| CA-242 (KU/ml) | 12.62 ± 0.45 | 2.15 ± 0.15 | 1.93 ± 0.41 | 0.656 |  | 14.50 ± 1.80 | 11.31 ± 3.56 | 3.67 ± 1.90 | 0.617 |
| AFP (ng/ml) | 3.53 ± 0.38 | 12.62 ± 2.97 | 14.52 ± 1.92 | 0.972 |  | 11.93 ± 0.98 | 12.75 ± 1.91 | 12.33 ± 3.22 | 0.932 |
| **characteristics** | **rs11911469** | | | |  | **rs1893650** | | | |
|  | **AA** | **AC** | **CC** | ***p***^†^ |  | **TT** | **TC** | **CC** | ***p***^†^ |
| CEA (ng/ml) | 16.53 ± 0.54 | 17.26 ± 1.44 | 23.41 ± 8.95 | 0.246 |  | 17.46 ± 0.74 | 15.41 ± 1.06 | 14.94 ± 0.98 | 0.235 |
| SF (ng/ml) | 89.03 ± 2.53 | 88.97 ± 0.82 | 90.42 ± 3.12 | 0.917 |  | 89.66 ± 0.53 | 87.83 ± 0.92 | 86.90 ± 2.25 | 0.108 |
| TNF (fmol/ml) | 1.05 ± 0.15 | 0.88 ± 0.01 | 0.87 ± 0.02 | 0.834 |  | 1.06 ± 2.49 | 0.88 ± 0.01 | 0.91 ± 0.01 | 0.786 |
| CA-50 (U/ml) | 7.43 ± 0.75 | 5.37 ± 1.15 | 4.08 ± 1.80 | 0.333 |  | 7.24 ± 0.81 | 6.80 ± 1.11 | 3.98 ± 1.06 | 0.448 |
| CA-199 (U/ml) | 41.51 ± 5.56 | 34.46 ± 5.30 | 28.08 ± 6.15 | 0.750 |  | 37.02 ± 4.75 | 48.68 ± 11.98 | 35.05 ± 7.31 | 0.512 |
| CA-242 (KU/ml) | 13.32 ± 1.72 | 16.02 ± 4.12 | 9.69 ± 4.73 | 0.729 |  | 14.19 ± 2.03 | 12.37 ± 2.63 | 15.68 ± 6.23 | 0.848 |
| AFP (ng/ml) | 12.48 ± 1.10 | 10.66 ± 0.65 | 11.64 ± 2.27 | 0.695 |  | 11.42 ± 0.66 | 14.30 ± 2.95 | 10.71 ± 2.02 | 0.339 |
| **characteristics** | **rs34904192** | | | |  | **rs928883** | | | |
|  | **AA** | **AG** | **GG** | ***p***^†^ |  | **AA** | **AG** | **GG** | ***p***^†^ |
| CEA (ng/ml) | 17.95 ± 2.12 | 16.87 ± 1.02 | 16.68 ± 0.75 | 0.880 |  | 17.38 ± 1.13 | 16.53 ± 0.87 | 16.99 ± 1.10 | 0.849 |
| SF (ng/ml) | 93.33 ± 2.03 | 89.44 ± 0.76 | 88.32 ± 0.58 | **0.027*** |  | 89.36 ± 0.94 | 88.67 ± 0.64 | 89.64 ± 0.87 | 0.624 |
| TNF (fmol/ml) | 2.79 ± 1.87 | 0.89 ± 0.01 | 0.89 ± 0.01 | **0.000*** |  | 0.90 ± 0.01 | 0.89 ± 0.01 | 1.27 ± 0.37 | 0.326 |
| CA-50 (U/ml) | 10.09 ± 4.38 | 5.59 ± 0.75 | 7.47 ± 0.88 | 0.166 |  | 10.06 ± 1.90 | 6.18 ± 0.73 | 5.81 ± 1.07 | **0.034*** |
| CA-199 (U/ml) | 21.74 ± 1.75 | 35.02 ± 6.88 | 44.47 ± 6.21 | 0.372 |  | 47.80 ± 14.05 | 38.53 ± 4.78 | 31.80 ± 7.13 | 0.425 |
| CA-242 (KU/ml) | 5.73 ± 1.18 | 12.86 ± 2.63 | 15.27 ± 2.18 | 0.372 |  | 12.66 ± 3.09 | 16.10 ± 2.59 | 8.58 ± 1.56 | 0.089 |
| AFP (ng/ml) | 18.64 ± 5.26 | 10.30 ± 0.46 | 12.55 ± 1.39 | 0.065 |  | 11.36 ± 1.34 | 12.21 ± 1.50 | 12.30 ± 1.25 | 0.923 |

**^†^ *p*-value was calculated from one-way anova analysis.**

***Bold values indicate statistical significance (p < 0.05).**
